# Supplementary material for: A single cell transcriptional roadmap of human pacemaker cell differentiation
Source: eLife. 2022 Oct 11;11:e76781. doi: 10.7554/eLife.76781 (PMC9553210; doi:10.7554/eLife.76781)
Supplement: Transparent reporting form [file elife-76781-transrepform1.docx]

***eLife’s* transparent reporting form**

We encourage authors to provide detailed information *within their submission* to facilitate the interpretation and replication of experiments. Authors can upload supporting documentation to indicate the use of appropriate reporting guidelines for health-related research (see [EQUATOR Network](http://www.equator-network.org/%20)), life science research (see the [BioSharing Information Resource](https://biosharing.org/)), or the [ARRIVE guidelines](http://www.plosbiology.org/article/info:doi/10.1371/journal.pbio.1000412) for reporting work involving animal research. Where applicable, authors should refer to any relevant reporting standards documents in this form.

If you have any questions, please consult our Journal Policies and/or contact us: [editorial@elifesciences.org](mailto:editorial@elifesciences.org).

**Sample-size estimation**

- You should state whether an appropriate sample size was computed when the study was being designed
- You should state the statistical method of sample size computation and any required assumptions
- If no explicit power analysis was used, you should describe how you decided what sample (replicate) size (number) to use

Sample size was not computed in advance. For qPCR experiments presented in figure 1, we wanted to demonstrate effects over a range of independent differentiation experiments as hiPSC cultures are prone to variation and heterogeneity. Hence, the choice of eight replicates (eight independent differentiations). In figure 2 (electrophysiology), we measured 12 cells per group (action potential characteristics) or 6 cells per group (response to ivabradine) from four independent differentiations. As single cell patch-clamp studies are labor intensive and low-throughput, experimental data is collected from cells spanning independent differentiations. For single cell RNA sequencing in figures 3, 4 and 7, we used cell populations from two independent differentiations for the end time point, which correlated well with one another. In figures 5 and 6, where the effects of certain treatments were tested, we evaluated four independent differentiations. Non-parametric statistical tests were used in all instances and specifics have been presented in each figure legends and/or the methods section.

Please outline where this information can be found within the submission (e.g., sections or figure legends), or explain why this information doesn’t apply to your submission:

**Replicates**

- You should report how often each experiment was performed
- You should include a definition of biological versus technical replication
- The data obtained should be provided and sufficient information should be provided to indicate the number of independent biological and/or technical replicates
- If you encountered any outliers, you should describe how these were handled
- Criteria for exclusion/inclusion of data should be clearly stated
- High-throughput sequence data should be uploaded before submission, with a private link for reviewers provided (these are available from both GEO and ArrayExpress)

Please outline where this information can be found within the submission (e.g., sections or figure legends), or explain why this information doesn’t apply to your submission:

The number of biological replicates (independent differentiations) is clearly mentioned in each figure legend. Technical replicates are only present in figure 2B-E (electrophysiology), where three cells each from four independent differentiations have been measured. Values for each cell measured have been presented in the source data file, which gives insight into both technical and biological variation. No outliers were encountered. Single cell RNA sequencing data is deposited in NCBI GEO repository under the accession number GSE189782. Reviewers can enter the token “uzqhiucyllqhdad” to access the data.

**Statistical reporting**

- Statistical analysis methods should be described and justified
- Raw data should be presented in figures whenever informative to do so (typically when N per group is less than 10)
- For each experiment, you should identify the statistical tests used, exact values of N, definitions of center, methods of multiple test correction, and dispersion and precision measures (e.g., mean, median, SD, SEM, confidence intervals; and, for the major substantive results, a measure of effect size (e.g., Pearson's r, Cohen's d)
- Report exact p-values wherever possible alongside the summary statistics and 95% confidence intervals. These should be reported for all key questions and not only when the p-value is less than 0.05.

Non-parametric tests were used for all analyses in the manuscript. For comparison between two groups (Fig.1, Fig.2, Fig.5, Fig.6), Mann-Whitney U Test was used. Paired comparison (Fig. 2E) was performed with Wilcoxon signed-rank test. Multiple comparison testing was done using Kruskal-Wallis test followed by Mann-Whitney U test. N numbers, dispersion and details of specific tests are presented in each figure legend.

Please outline where this information can be found within the submission (e.g., sections or figure legends), or explain why this information doesn’t apply to your submission:

(For large datasets, or papers with a very large number of statistical tests, you may upload a single table file with tests, Ns, etc., with reference to sections in the manuscript.)

**Group allocation**

- Indicate how samples were allocated into experimental groups (in the case of clinical studies, please specify allocation to treatment method); if randomization was used, please also state if restricted randomization was applied
- Indicate if masking was used during group allocation, data collection and/or data analysis

Two distinct directed differentiation protocols were used to generated sinoatrial nodal and ventricular cells, which was confirmed by using molecular and electrophysiological characterization presented in figure 1 and 2. Thus, group allocation is justified.

Please outline where this information can be found within the submission (e.g., sections or figure legends), or explain why this information doesn’t apply to your submission:

**Additional data files (“source data”)**

- We encourage you to upload relevant additional data files, such as numerical data that are represented as a graph in a figure, or as a summary table
- Where provided, these should be in the most useful format, and they can be uploaded as “Source data” files linked to a main figure or table
- Include model definition files including the full list of parameters used
- Include code used for data analysis (e.g., R, MatLab)
- Avoid stating that data files are “available upon request”

Source files have been provided for electrophysiology data presented in figure 2 and figure 7.

Single cell RNA sequencing data, which is the basis for Figures 3 – 7 is deposited in NCBI GEO repository under the accession number GSE189782. Reviewers can enter the token “uzqhiucyllqhdad” to access the data.

R scripts used for analysis of single cell RNA sequencing data are available on Github (<https://github.com/wiesingera/transcriptional_roadmap_hiPSC-SANCM>) and can be accessed via <https://gitfront.io/r/user-5927993/61R3PyCthVFw/transcriptional-roadmap-hiPSC-SANCM-gitfront/>.

We have also provided differentially expressed gene lists of all clusters described in figure 3, 4, 5, 6 and 7.

Please indicate the figures or tables for which source data files have been provided:
